# Changes in Frontal Band Power and Pupil Diameter Predict Performance During Motor Imagery Neurofeedback

**DOI:** 10.1111/psyp.70380

**Published:** 2026-08-17

**Authors:** Agustina Fragueiro, René‐Paul Debroize, Claire Cury

**Affiliations:** ^1^ Univ Rennes, Inria, CNRS, Inserm, IRISA UMR 6074, EMPENN—ERL U 1228 Rennes France; ^2^ Dipartimento di Psicologia Università G. d'Annunzio di Chieti‐Pescara Chieti Italy; ^3^ Istituto di Tecnologie Avanzate Biomediche Università G. d'Annunzio di Chieti‐Pescara Chieti Italy

**Keywords:** alpha desynchronization, cognitive engagement, neurofeedback, pupil diameter, sustained attention, theta synchronization

## Abstract

Neurofeedback (NF) offers potential for cognitive enhancement and brain rehabilitation, yet a substantial proportion of individuals fail to acquire self‐regulation of brain activity. Task engagement, strongly linked to attentional focus and arousal, is a critical determinant of NF success. Frontal theta and alpha band oscillations, as well as pupil diameter and skin conductance responses are known to reflect attentional control, cognitive workload and arousal, all of which influence task performance. The present study investigates the relationship between frontal theta and alpha power, pupil diameter and skin conductance with performance during motor imagery NF training. By examining these multimodal physiological markers, we aimed at identifying reliable indicators of cognitive engagement and predictors of NF performance. Our findings indicate that fluctuations in NF performance are systematically associated with distinct psychophysiological changes within individuals, but also globally at a group level. Frontal theta synchronization, alpha desynchronization and pupil diameter were strongly related to NF performance. Notably, alpha desynchronization and pupil diameter emerged as significant predictors of the individual performance level, with stronger frontal alpha desynchronization and increased pupil diameter predicting higher performance. Our results suggest the potential of theta synchronization, alpha desynchronization and pupil diameter as dynamic markers of engagement and performance during NF sessions.

## Impact Statement

This study identifies multimodal physiological markers that track and predict individual performance during motor imagery neurofeedback. By establishing these real‐time indicators of cognitive engagement, our findings lay the groundwork for personalized neurofeedback systems that dynamically adapt to the user's cognitive state, directly addressing the widespread problem of neurofeedback inefficacy in rehabilitation.

## 1 Introduction

Neurofeedback (NF) is a technique that involves providing individuals with real‐time feedback on their neural activity, enabling them to learn how to self‐regulate brain function (Sitaram et al. 2017). As a non‐invasive and potentially long‐lasting intervention, NF holds significant promise in promoting neuroplasticity (Loriette et al. 2021). It has emerged as a valuable tool for brain rehabilitation, demonstrating the capacity to correct maladaptive neural patterns associated with a range of brain disorders, including psychiatric (Arns et al. 2017) and neurodevelopmental disorders (Holtmann et al. 2011), as well as for post‐stroke rehabilitation (Le Franc et al. 2022; Lioi, Butet, et al. 2020; Renton et al. 2017).

NF is most commonly implemented using real‐time electroencephalography (EEG). It has been studied for several decades (Marzbani et al. 2016) and it continues to be widely applied in both rehabilitation and cognitive enhancement contexts (Bai et al. 2020). In motor rehabilitation, NF protocols often incorporate motor imagery tasks, as imagining and executing movements activate overlapping brain regions—particularly during kinesthetic motor imagery (Solodkin et al. 2004). Moreover, it has been shown that NF can enhance the efficacy of motor imagery training, in terms of eliciting brain patterns relevant to the task (Bagarinao et al. 2018; Zich et al. 2015). Specifically, motor imagery is associated with an amplitude reduction of brain oscillations from sensorimotor cortical areas (Cheyne 2013). Thus, when imagining or executing movements sensorimotor rhythms of central channels desynchronize in the 8 to 30 Hz frequency range (Pfurtscheller and Neuper 2001). Recent findings have revealed the potential of motor imagery NF in stroke rehabilitation (Cervera et al. 2018) to stimulate neural plasticity and support functional improvement (Grosse‐Wentrup et al. 2011).

Despite its promise, NF is not always effective. Approximately 38% of participants across studies fail to modulate their brain activity effectively (Haugg et al. 2021)—a phenomenon referred to as the NF inefficacy problem (Alkoby et al. 2018). Motivation and attention have emerged as key predictors of NF success (Haugg et al. 2021; Hammer et al. 2012). Low motivation can impair attention, potentially leading to disengagement from the task and the risk of being classified as a “non‐responder” (Kadosh and Staunton 2019). To improve training outcomes, it has been recommended to monitor participants' motivation levels (Sorger et al. 2019) and tailor NF protocols to individual needs (Alkoby et al. 2018). Specifically in post‐stroke NF rehabilitation, individualized and adaptive approaches have been linked to improved efficacy (Le Franc et al. 2022).

Sustained attention—the ability to maintain focus on task‐relevant stimuli over time—is crucial for successful performance but is inherently effortful and susceptible to lapses (i.e., disengagement from the task). Performance has been steadily associated with arousal level (Yerkes and Dodson 1908), with both hypo‐ and hyper‐arousal linked to off‐task mental states such as mind‐wandering or distraction (Unsworth and Robison 2017, 2018). Optimal cognitive engagement—defined as the allocation of limited mental resources to a task (Kahneman 1973) – and successful performance tend to occur at moderate arousal levels (Aston‐Jones and Cohen 2005). Thus, maintaining on‐task focus of attention, in combination with an adequate arousal level, are important determinants of cognitive engagement and, consequently, a successful performance (Lenartowicz et al. 2013).

EEG studies have consistently linked attentional states and cognitive workload to changes in oscillatory brain activity. Sustained attention is thought to be supported by fronto‐medial theta oscillations and modulated through selective excitation and inhibition via gamma and alpha activity, respectively (Clayton et al. 2015). Increases in cognitive demand are typically associated with elevated theta power and reduced alpha power, particularly at frontal‐central sites (Magosso et al. 2021; Vidulich and Tsang 2012). However, this relationship appears to be non‐linear. Puma et al. (2018) reported that theta and alpha power, for the frontal channel Fz, increased with task demand up to a ceiling point, beyond which performance declined. Notably, the highest task performance was associated with the lowest spectral power in both theta and alpha bands, in line with earlier work showing a negative correlation between theta power and performance (Klimesch 1999; Kramer 1991). The role of alpha oscillations in cognitive performance remains complex, as their modulation appears to reflect distinct neural mechanisms. While some studies have reported an alpha power decrease in central and parietal regions during task execution and mental effort (Fairclough and Venables 2006; Fukuda et al. 2015), others have observed that central and parietal alpha increases in contexts requiring the inhibition of irrelevant information (Pope et al. 1995; Sauseng et al. 2009). In general, alpha desynchronization has been proposed to facilitate relevant sensory processing (Capilla et al. 2014; Fukuda et al. 2015; Rihs et al. 2007), while higher alpha power has been linked to disengagement or mind‐wandering (Compton et al. 2019). Both theta and alpha rhythms, through distinct but interacting mechanisms, are sensitive markers of attentional control, cognitive workload, and the dynamic balance between externally and internally guided attention (Ceh et al. 2020, 2021; Magosso et al. 2021).

Beyond EEG, eye‐tracking and skin conductance have been widely used to assess physiological correlates of attention and arousal. In particular, specific EEG indicators have been identified in conjunction with ocular measures—a well‐established proxy for attentional focus. Both EEG alpha activity and pupil diameter are considered time‐sensitive markers of internal cognitive demand and are thought to reflect a neurophysiological gating mechanism that filters out irrelevant sensory input to protect internal cognitive processes (Ceh et al. 2020, 2021). Combined eye‐tracking and EEG measures have also been associated with vigilance levels (Bodala et al. 2016). In addition, a recent study has shown that the combination of various eye‐tracking and skin conductance features was able to predict performance in several cognitive tasks (Fragueiro et al. 2026). In the present study, we focus specifically on pupil diameter, as it has been robustly linked to cognitive load (Piquado et al. 2010), task performance (van den Brink et al. 2016), fatigue and engagement (Hopstaken et al. 2016), and mind‐wandering (Groot et al. 2021). Additionally, the phasic component of skin conductance signal—characterized by rapid fluctuations—has been associated with arousal (Lane et al. 1999) and is known to respond quicker to cognitive and emotional stimuli than the slower tonic component (Critchley 2002).

The present study aimed at investigating the relationship between pupil diameter, skin conductance, frontal theta and alpha band power, with NF performance during an EEG‐based motor imagery NF session in adult participants. Our objective was to explore whether these physiological features can serve as reliable indicators for predicting changes in performance during neurofeedback training.

## 2 Method and Materials

### 2.1 Participants

Twenty‐four right‐handed volunteers (12 females, age range = 20–60 years old) reporting normal vision participated in our study after signing informed consent written following guidelines of the Open Brain Consent (Bannier et al. 2021). This study has been accepted by the Comité Opérationnel d'Évaluation des Risques Légaux et Éthiques (COERLE) of Inria, the Ethics Review Board at Inria complying with the European General Data Protection Regulation (approval ID 2024–04).

### 2.2 Procedure

**FIGURE 1:**
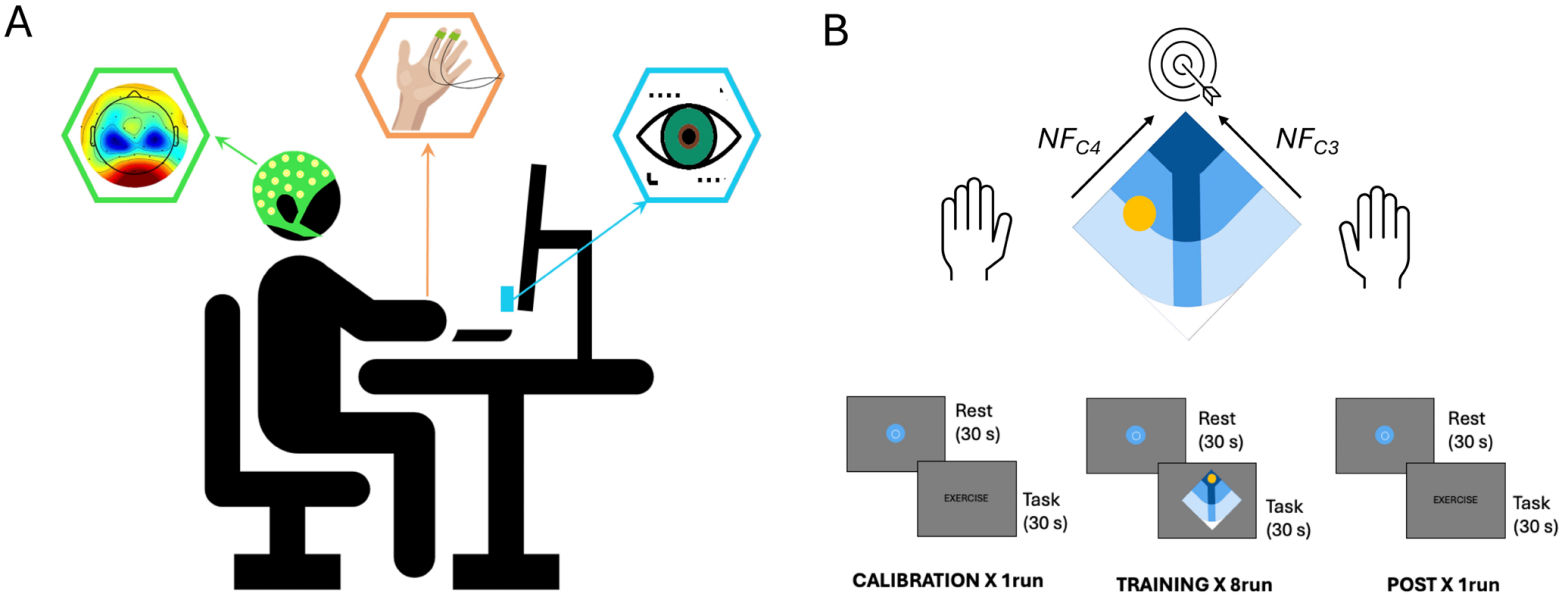
(A) Multimodal set up registering simultaneously EEG, skin conductance and eye‐tracking. (B) Neurofeedback visual metaphor and experimental paradigm including calibration, training and post runs.

Participants completed a 10‐min EEG‐based NF session while sitting in front of a screen with 1920 × 1080 display resolution in a dark room, while simultaneously recording: (1) EEG, using a Brain Products actiCAP set of 32 active electrodes and an actiChamp amplifier; (2) eye‐tracking, using a screen‐based eye‐tracker Tobii Pro X2‐120; (3) skin conductance from the index and middle fingers using a BrainVision galvanic skin response set (Figure 1A).

### 2.3 Neurofeedback

Participants were instructed to perform a kinesthetic motor imagery task with both hands simultaneously. They were presented with a visual representation (from now on called “visual metaphor”), a yellow ball moving inside a blue square rotated 45 degrees (Figure 1B), depicting event‐related desynchronization (ERD) of the C3 and C4 electrodes. The participant's goal was to keep the ball in the upper corner of the square, corresponding to a simultaneous motor imagery of both hands. Specifically, participants were instructed to move the ball on the screen by imagining hand movements. They were also informed that the task is generally performed more successfully when focusing on the kinesthetic sensation associated with the imagined hand movements. They were informed that if their focus was more directed to the right or the left hand, the ball would move to the respective corner of the square. The NF session consisted of one calibration, eight training runs and one post run. Each run was composed of one rest block (30 s) followed by one motor imagery task block (30 s).

The Cz electrode was used as the reference. The OpenVibe software (Renard et al. 2010) was used for real‐time signal processing. EEG data were segmented into overlapping 2‐s epochs using a sliding window with a step size of 250 ms. The neurofeedback display was updated every 250 ms. Two discrete Laplacian spatial filters Lap(C3) and Lap(C4) were estimated around C3 and C4 channels. Each Laplacian filter uses a weight of 8 for the central channel and a weight of −1 for the surrounding channels (i.e., FC5, FC1, CP5, CP1 surround C3, and FC6, FC2, CP6, CP2 surround C4). Other channels were ignored during the NF session. The power of Lap(C3) and Lap(C4), denoted as Bp_*C*3_, Bp_*C*4_, was computed in the 8–30 Hz frequency band. Values of Bp_*C*3_ and Bp_*C*4_ were continuously sent to a dedicated program via the LSL library to compute neurofeedback scores and display the ball in the right position on the metaphor. Neurofeedback scores (denoted NF_*C*3_ and NF_*C*4_) were computed the same way, with NF_*C*3_(t) = [Bp_*C*3_(ref) − Bp_*C*3_(t)]/Bp_*C*3_(ref) where Bp_*C*3_(ref) = med(Bp_*C*3_(rest[10:20])), the median of the band power during the 10 s central interval of the last rest block (Lioi, Cury, et al. 2020). The goal target (corresponding to the higher corner in dark blue, Figure 1B) was set for each hand independently based on the 70th percentile of the scores achieved during calibration. If this score achieved during calibration was lower than 0.15, the target (i.e., maximal value) was set to 0.15 to avoid a very low threshold (thus, close to 0 or negative values). To represent scores in the metaphor, a normalization from 0 to 1, using the above‐mentioned threshold, was applied to compute the position of the ball within the square. Any score below 0, indicating absence of ERD, was capped to 0, the minimal value of NF scores (lower corner of the square).

### 2.4 EEG Offline Processing

In addition, we processed data offline with EEGLAB v2025.0.0 running on Matlab R2025. Cz was estimated and reinserted in the dataset as it was the original reference following the EEGLAB pipeline to re‐reference EEG data.^1^ EEG was band pass (1–40 Hz) filtered. Bad channels were automatically rejected based on kurtosis (threshold = 5). In agreement with standard pipelines (Delorme and Makeig 2004) and with previous studies on frontal theta and alpha band power (Puma et al. 2018), data were re‐referenced to average. Subsequently, signals were decomposed using an ICA and independent components were automatically labeled with ICLabel 1.5 (Pion‐Tonachini et al. 2019). Components not identified as brain activity with at least 50% probability were rejected after visual inspection. Data were divided based on the start of the rest and task events setting time limits between 1 and 29 s. So, two datasets were created separately for each subject (i.e., rest, task). Each segment (corresponding to each NF run, 8 in total) was epoched in 2‐s overlapping temporal windows sliding every 1 s.

For further analyses on frontal band power and cognitive workload (Puma et al. 2018), a Laplacian filter was applied on Fz, by subtracting the mean of the neighbor channels F3 and F4. A Hanning window was applied to each segment to reduce spectral leakage, and power spectral density computed via Fast Fourier Transformation. Finally, Fz power in the theta (4–8 Hz) and alpha (8‐12 Hz) bands was extracted for each segment corresponding to each NF run (8 in total). A natural log transformation was applied to normalize the power values and reduce skewness. Finally, to obtain event‐related synchronization (ERS) for the theta band, power of the immediately previous rest (R‐1) block was subtracted from the task (T) band power for the channel Fz: $\mathrm{ER}S_{\theta }\left(\mathrm{Fz}\right)=Bp_{\theta }\left(T\right)-Bp_{\theta }\left(R-1\right).$ On the contrary, to observe frontal event‐related desynchronization (ERD) for the alpha band, power of the task block T was subtracted from the immediately previous rest block R‐1: $\mathrm{ER}D_{\alpha }\left(\mathrm{Fz}\right)=Bp_{\alpha }\left(R-1\right)-Bp_{\alpha }\left(T\right).$

### 2.5 Skin Conductance

A BrainVision galvanic skin response set was used to acquire electrodermal activity from the index and middle fingers. After downsampling to 10 Hz, skin conductance (SC) responses were estimated through a continuous decomposition analysis using MATLAB toolbox Ledalab (Benedek and Kaernbach 2010). Integrated phasic driver activity (ISCR), which corresponds to the area of the phasic driver within each temporal window, was extracted by setting 10 s consecutive temporal windows. To account for individual variability, signal was *Z*‐scored for each participant using the whole training session. Finally, data were averaged by block for the further analyses.

### 2.6 Eye‐Tracking

Eye activity was recorded with a 120 Hz acquisition rate using the Tobii Pro standard calibration procedure, and the output was saved using its Software Development Kit (SDK). When pupil detection was judged as valid for both eyes according to the SDK validity codes, pupil diameter (PD) was averaged between both eyes. To account for individual variability, signal was *Z*‐scored for each participant using the whole training session. Finally, PD was averaged by block for further analyses.

### 2.7 Statistical Analysis

We first aimed at validating NF results offline and testing participant's learning within the session (Section 2.7.1). We compared NF scores computed online during the NF session, but also, we compared band power for all channels between rest and task conditions after offline processing of EEG data. In addition, we explored changes in skin conductance and pupil diameter along all the session (Section 2.7.2). Subsequently, we investigated the relationship between NF scores (computed online) and physiological changes associated with attention and arousal, which, to our knowledge, may serve as indices of cognitive engagement (Section 2.7.3). To do this, we first conducted repeated measures correlations (Bakdash and Marusich 2017) between NF scores and frontal theta ERS ($\mathrm{ER}S_{\theta }$), alpha ERD ($\mathrm{ER}D_{\alpha }$), pupil diameter (PD) and phasic skin conductance responses (ISCR). With the same aim, we also classified NF performance in levels (i.e., low, middle and high) and observed the relationship between the same physiological features and performance levels. Finally, we tested the predictive value of these physiological features on NF performance with a logistic regression, for categorical data, and a linear regression, for continuous data (Section 2.7.4).

#### 2.7.1 Offline Validation of NF Data

Statistical analyses were conducted on 23 participants, due to artifacts in the EEG signal of one participant. To analyze NF performance, we considered three NF scores: NF_*C*3_, NF_*C*4_ and a total NF score that was defined as $NF_{\text{total}}={\mathrm{NF}}_{C3}+{\mathrm{NF}}_{C4}-\mathrm{abs}\left({\mathrm{NF}}_{C3}-{\mathrm{NF}}_{C4}\right)$. To test learning during the NF session, paired sample *t*‐tests were conducted between calibration and post runs for NF_*C*3_, NF_*C*4_, and NF_total_ on 20 subjects due to recording problems in the post block for 3 subjects. All further analyses were conducted on 23 subjects but runs with outlier scores distancing from 3 standard deviations from the group average, were excluded (5 runs, corresponding to 4 different participants, were excluded). To observe progression in the NF scores along the training session, one‐way ANOVAs were conducted independently for NF_*C*3_, NF_*C*4_ and NF_total_, including NF scores as dependent variable and order (runs from 1 to 8) as independent factor.

To compare power spectrum between the rest and task conditions during training, after Fast Fourier Transformation and band power extraction, paired *t*‐tests were conducted on all channels independently (32 channels) for the bands of frequency theta (4–8 Hz) and alpha‐beta (8–30 Hz). To correct for multiple comparisons the significance threshold was set at alpha = 0.05/32.

#### 2.7.2 Skin Conductance and Pupil Diameter

Analyses on skin conductance using the ISCR feature were conducted on 19 participants due to problems in signal recording in 5 participants. Analyses on pupil diameter were conducted on 17 participants due to a change in the screen luminosity for the first 6 participants (4 of them correspond to the same subjects excluded for ISCR problems). Runs with outlier values distancing from 3 standard deviations from the group average were excluded (3 runs for ISCR and 1 for PD were excluded, all of them corresponding to different subjects). To observe the trend on ISCR and PD during the session, two independent two‐way ANOVAs were conducted including condition (rest vs. task) and order (runs from 1 to 8) as independent factors and ISCR and PD as dependent variables. *p*‐values corresponding to post hoc tests were Bonferroni corrected for multiple comparisons.

#### 2.7.3 Relationship Between NF Scores and Physiological Features

To study the relationship between NF scores as a continuum and the extracted physiological features, repeated measures correlations (Bakdash and Marusich 2017)—to account for the non‐independence of data within participants—were computed between NF_total_ and ERS_θ_, ERD_ɑ_, PD, and ISCR considering the 8 runs for each participant (8 runs × 23 subjects). Runs with outlier values distancing from 3 standard deviations from the group average, were excluded (2 runs for $\mathrm{ER}S_{\theta }$, 3 runs for $\mathrm{ER}D_{\alpha }$, and 2 runs for both—all of them corresponding to 4 different participants). To correct for multiple comparisons (i.e., 4 tests) the significance threshold was set at alpha = 0.05/4.

Finally, in order to get levels of NF performance, runs were divided based on the NF_total_. All NF_total_ below 0 were classified as low performance, while NF_total_ above 0 were classified as middle (below the mean value of the remaining scores) and high (above the mean of the remaining scores) performances. We conducted two different classifications. In the first classification, the threshold between middle and high performance was set within each subject individually (based on the individual average of the scores above 0), so we could observe changes/improvements in performance within each participant. In the second classification, a common threshold was set for all the group, to get middle and high performances classified across all participants (based on the group average of all NF_total_ > 0). For each classification, four independent one‐way ANOVAs were conducted for ERS_θ_, ERD_ɑ_, PD, and ISCR as dependent variables, including always NF performance level (low, middle, high) as independent factor. While the first classification allowed us to observe changes in performance within each subject individually; the second classification allowed us to investigate a general trend in the relationship between performance and physiological changes. *p*‐values corresponding to post hoc tests were Bonferroni corrected.

#### 2.7.4 Predicting NF Performance With Physiological Features

In order to investigate if the extracted physiological features may be used to predict a high vs. a low level of performance, we conducted a logistic regression including the individual NF level (i.e., only high and low levels, to stress the differences) for each run independently as dependent variable, and all the extracted physiological features (i.e., ERS_θ_, ERD_ɑ_, PD, and ISCR) as independent factors.

Finally, to further expand our results and test the relationship between these features and NF performance as a continuous score, we tested a linear regression model including NF_total_ as dependent variable and ERS_θ_, ERD_ɑ_, PD, and ISCR as predictors.

## 3 Results

### 3.1 Offline Validation of NF Data

After the exclusion of one participant due to artifacts in the EEG signal, the average score before normalization obtained across participants over all runs with the real time computation were 0.10 (min = −0.94, max = 0.72) for C3 and 0.12 (min = −1.23, max = 0.75) for C4. The average NF_total_ was 0.03 (min = −2.48, max = 1.34). These results evidenced an important variability in NF scores across participants.

**FIGURE 2:**
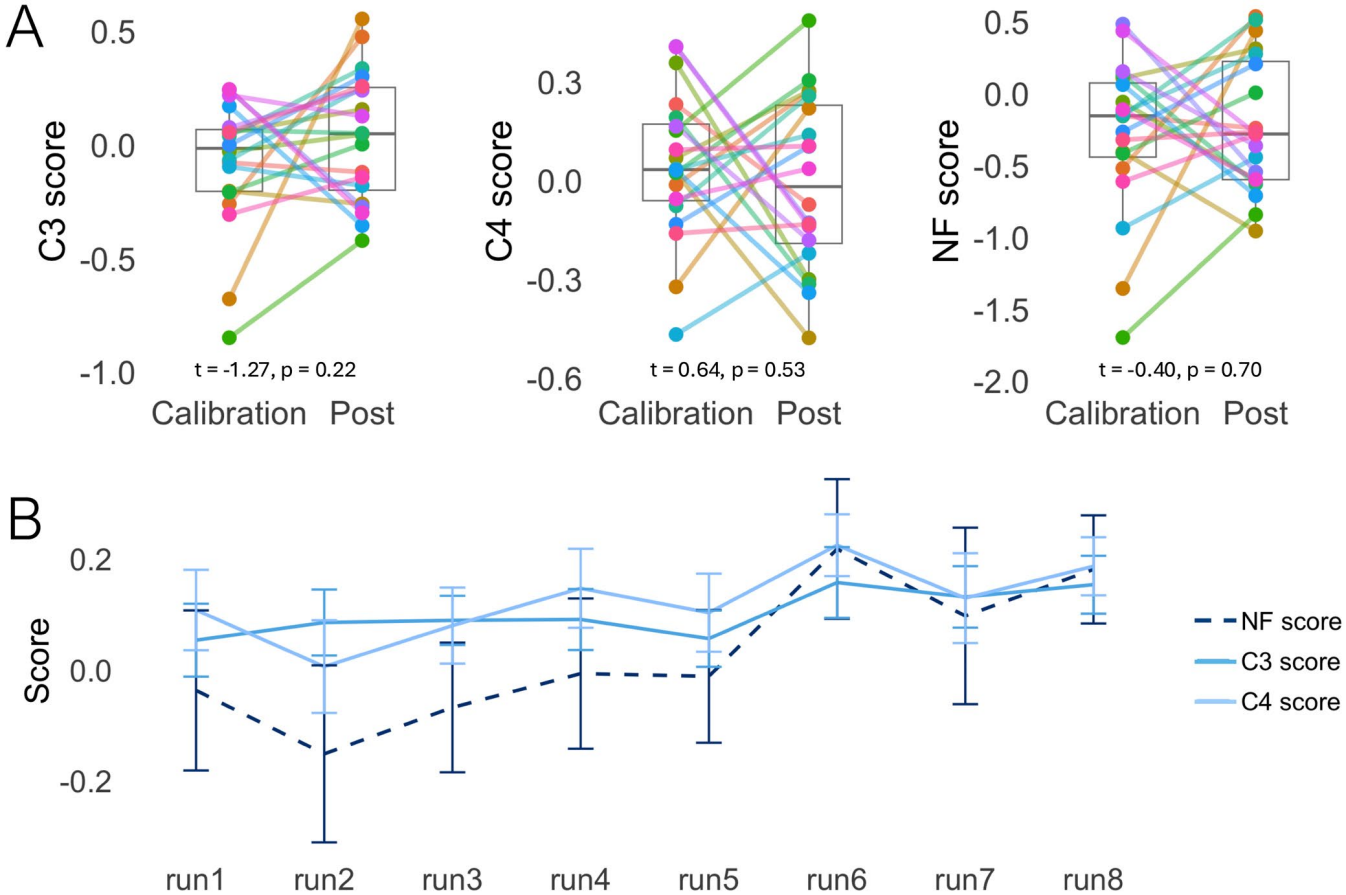
(A) Neurofeedback scores for calibration and post‐training runs for each subject individually in channel C3 (NF_*C*3_), C4 (NF_*C*4_) and the total NF score (NF_total_). For each condition, boxplots show the median of the scores, the 75th and the 25th percentiles. Whiskers extend to the lowest and highest points within 1.5 interquartile range. (B) Mean and standard error of neurofeedback scores for each training run.

No significant difference was observed between calibration and post runs for any channel (NF_*C*3_, NF_*C*4_), nor for NF_total_ (Figure 2A). Although there is a slight improvement in the NF scores along the session (Figure 2B), the one‐way ANOVA conducted along the 8 runs was not significant for NF_*C*3_ or NF_*C*4_, nor for NF_total_.

**FIGURE 3:**
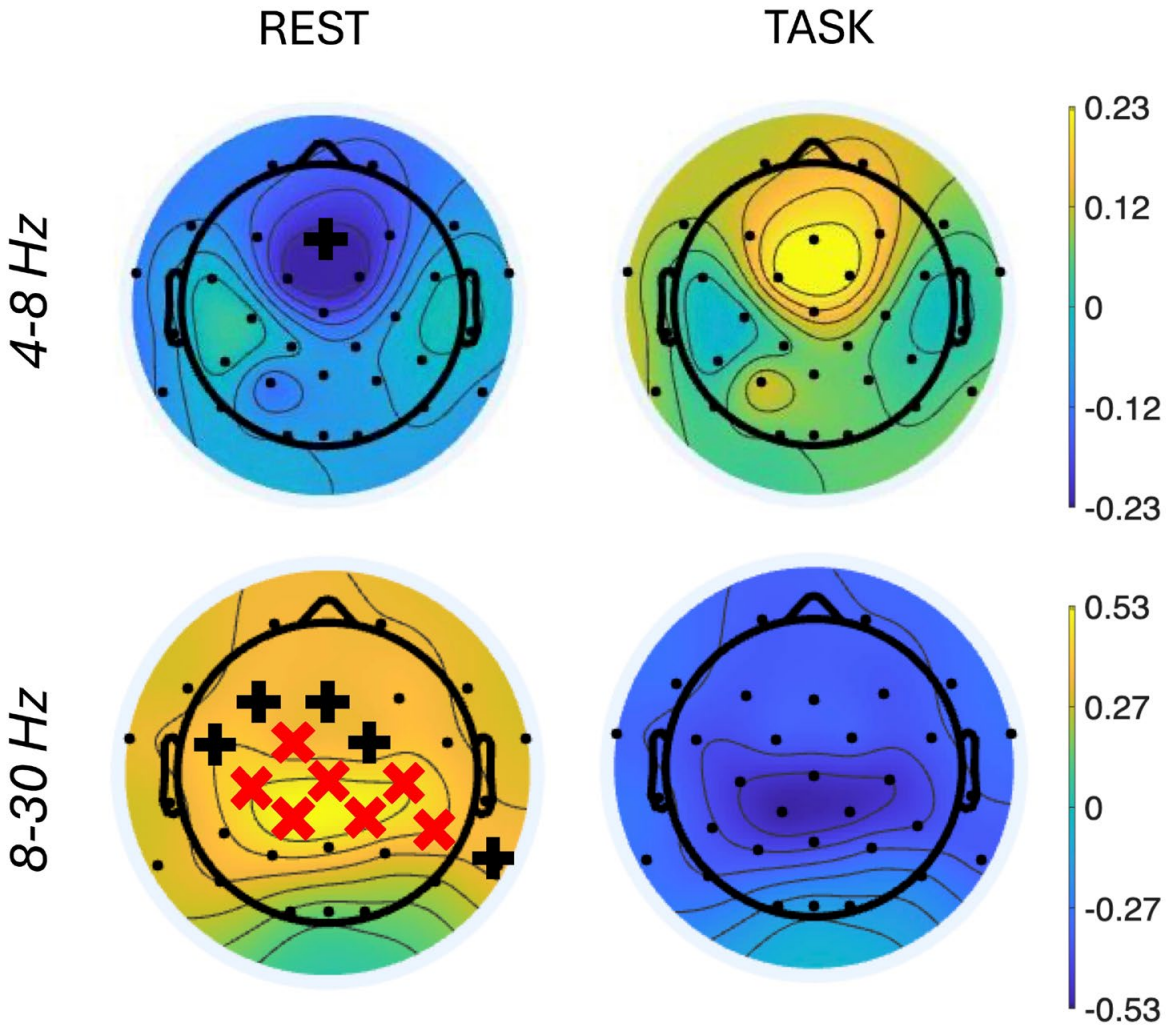
Spectroplots for alpha and theta bands of frequency comparing rest vs. task blocks of all the training runs. + *p* < 0.002, × *p* < 0.001.

Paired *T*‐tests conducted between rest and task conditions of the NF training for all 32 channels evidenced significant differences (at a significance threshold of alpha = 0.0016) in the theta band for Fz (*t*_(22)_ = −3.653, *p* = 0.001), and a not significant trend for FC1 (*t*_(22)_ = −3.599, *p* = 0.002) and FC2 (*t*_(22)_ = −3.397, *p* = 0.003), and in the alpha‐beta band for C3, C4, Cz, CP1, CP2, CP6, FC1 (all *ps* < 0.001), but also for Fz, F3, FC2, FC5 and TP10 (all *ps* < 0.002) (Figure 3). As expected, alpha‐beta desynchronization is observed in bilateral sensorimotor central channels corresponding to the motor imagery activity of both hands, while frontal theta synchronization is thought to be related to mental workload linked to the task execution.

### 3.2 Skin Conductance and Pupil Diameter

**FIGURE 4:**
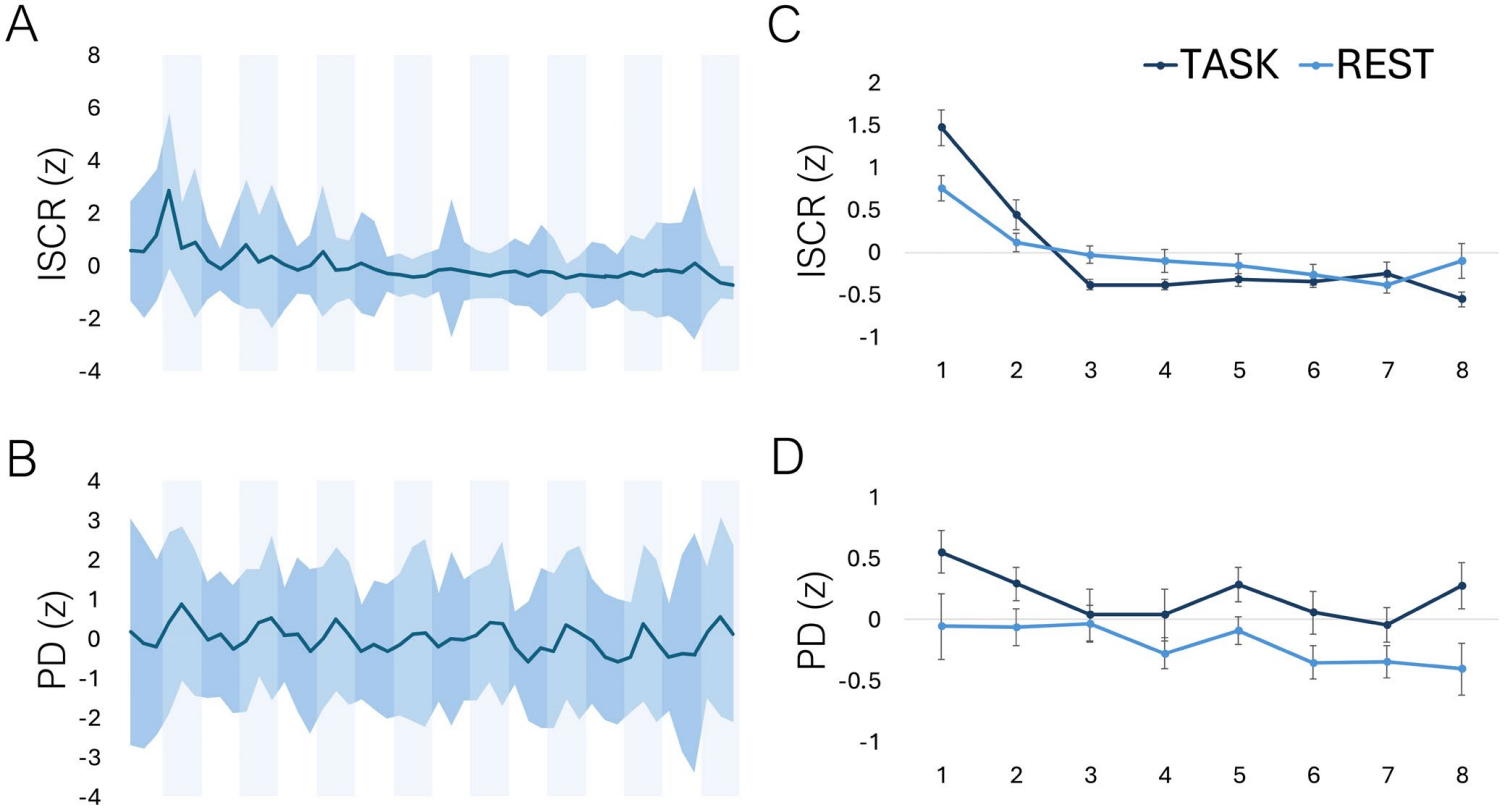
Mean (A) Integrated phasic driver activity of skin conductance responses (ISCR), and (B) pupil diameter (PD) for all participants averaged in 10 s temporal windows along the eight runs of the neurofeedback session. Each run is composed of one rest (shadowed in white) and one task block (shadowed in light blue). The shadowed blue area depicts 2 standard deviations from the group mean. (C) Average ISCR and (D) PD for all participants during task and rest conditions along the 8 runs of the NF session.

The two‐way ANOVA conducted on skin conductance (ISCR), including condition (rest vs. task) and order (i.e., from 1 to 8) as independent factors, evidenced only a significant effect of order (*F*_(7,288)_ = 30.061, *p* < 0.001, η^2^ = 0.40), and a significant interaction (*F*_(7,288)_ = 4.552, *p* < 0.001, η^2^ = 0.06). Bonferroni corrected post hoc comparisons confirmed that ISCR for the 1st and 2nd runs was significantly higher compared to all the other runs (all *ps* < 0.01) (Figure 4A,C).

The same analysis was done for PD. We observed only a significant effect of condition (*F*_(1,239)_ = 24.990, *p* < 0.001, η^2^ = 0.09); no significant effect was observed for order, nor interaction. Specifically, Bonferroni corrected post hoc comparisons showed that PD in the rest conditions was significantly smaller compared to the task (*t*_(239)_ = −4.999, *p* < 0.001) (Figure 4B,D).

### 3.3 Relationship Between NF Scores and Physiological Features

**FIGURE 5:**
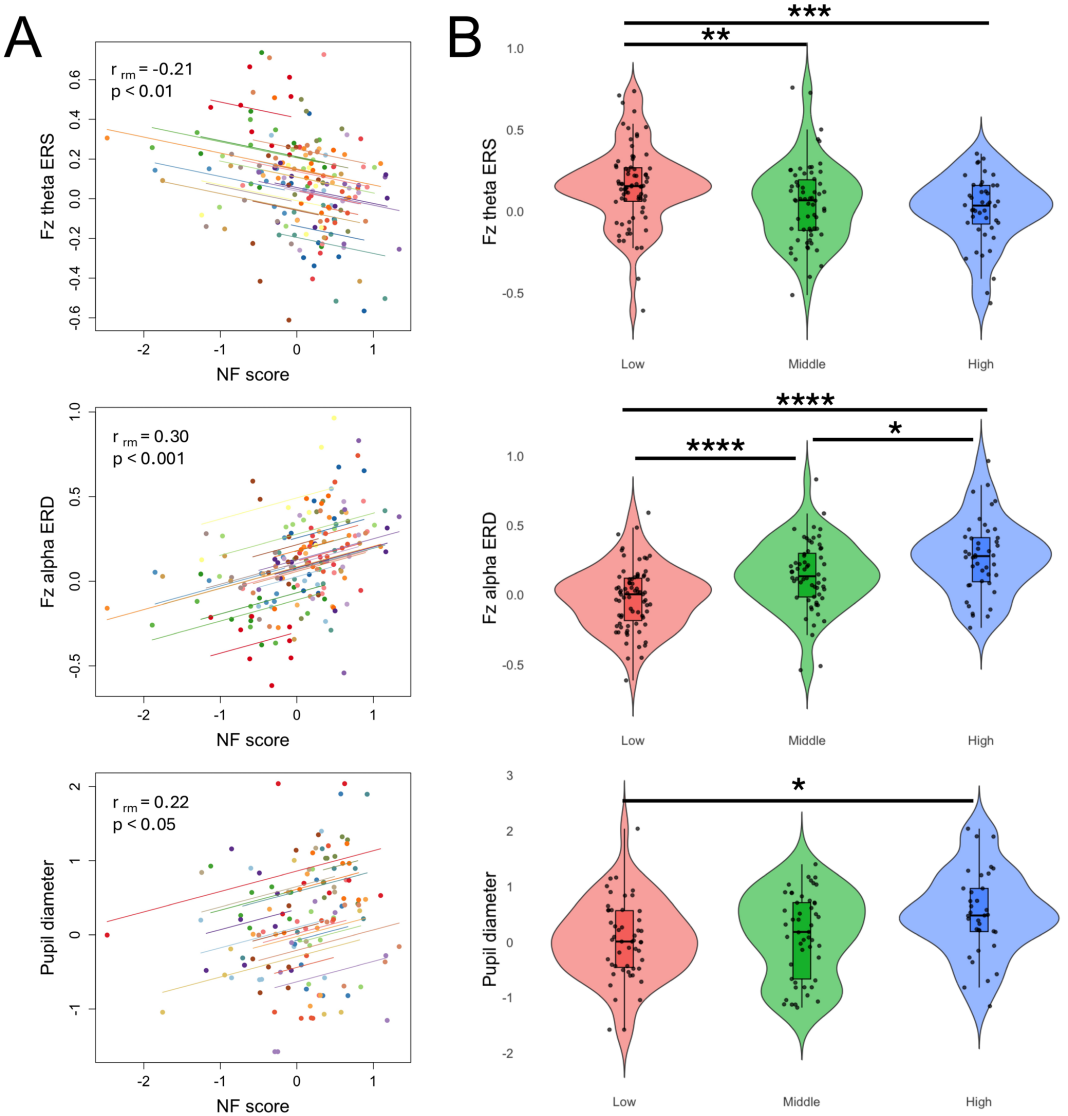
(A) Dispersion plots and repeated measures correlations between NF_total_ scores and frontal theta ERS, alpha ERD, and pupil diameter. (B) Violin plots depicting frontal theta ERS, alpha ERD and pupil diameter divided by NF level (i.e., low, middle, high) set for each subject individually. Bonferroni corrected *p* = * < 0.05, ** < 0.01, *** < 0.005, **** < 0.001.

We first investigated the relationship between NF_total_ and all physiological features along the NF session including the 8 runs for each subject. Significant repeated measures correlations (at a significance threshold of alpha = 0.0125) were observed between NF_total_ and frontal (i.e., Fz) ERS_θ_ (*r*_*rm*_ = −0.21, *p* = 0.009) and ERD_ɑ_ (*r*_*rm*_ = 0.30, *p* < 0.001). Thus, lower theta synchronization during the task was associated with higher NF scores, whereas greater alpha desynchronization was associated with higher NF scores. In addition, an almost significant trend for a positive correlation between PD and NF_total_ (*r*_*rm*_ = 0.22, *p* = 0.017) was observed, so bigger PD was associated with higher NF_total_ (Figure 5A). The correlation between NF_total_ and ISCR was not significant.

In a second step, we divided all runs in three levels based on NF_total_ to obtain low, middle and high performance for each participant independently. We aimed at emphasizing individual changes/improvements in performance along the session, and testing differences in individual physiological features (i.e., ERS_θ_, ERD_ɑ_, ISCR and PD) associated with them. After classifying all NF_total_ below 0 as low performance, a threshold was set between middle and high performance at an individual level, reflecting important interindividual differences: while 3 participants got all scores within a low level, 2 participants achieved no score below 0. After classification, in total 75 runs corresponded to a low‐level performance, 58 to middle and 46 to high. The low level corresponded to a mean NF_total_ across subjects of −0.52 (SD = 0.49), the middle level to 0.27 (SD = 0.17), and the higher level to 0.67 (SD = 0.27).

One‐way ANOVAs including physiological features as dependent variable and the NF level as independent factor were significant for frontal ERS_θ_ (*F*_(2,173)_ = 7.508, *p* < 0.001, η^2^ = 0.08), ERD_ɑ_ (*F*_(2,173)_ = 22.187, *p* < 0.001, η^2^ = 0.20) and PD (*F*_(2,128)_ = 4.489, *p* = 0.013, η^2^ = 0.07), not for ISCR. Bonferroni corrected post hoc comparisons evidenced that runs with lower performance presented higher ERS_θ_ compared to middle (*t*_(173)_ = 3.086, *p* = 0.007) and high performance (*t*_(173)_ = 3.392, *p* = 0.002), as well as smaller PD (*t*_(128)_ = 2.937, *p* = 0.011) compared to runs with high performance. Significant differences were observed for ERD_ɑ_ across all levels: ERD_ɑ_ was lower for runs with low performance compared to middle (*t*_(173)_ = −4.069, *p* < 0.001) and high (*t*_(173)_ = −6.460, *p* < 0.001) levels, and for runs with middle comparing to those with high performance (*t*_(173)_ = −2.549, *p* = 0.034) (Figure 5B).

We also investigated if the same tendency can be observed at a general level among participants. Thus, we investigated whether, when setting threshold based on the group average NF_total_, runs with higher or lowest performances have the same relationship with these physiological features as the one observed at an individual level. The same 75 runs with a score below 0 were classified as low, 57 as middle and 47 as high‐level performance. For the whole group, the middle level corresponded to a mean NF_total_ of 0.23 (SD = 0.11) and the higher level to 0.71 (SD = 0.23).

Once again, when classifying performance setting shared thresholds for the whole group, one‐way ANOVAs were significant for ERS_θ_ (*F*_(2,173)_ = 8.478, *p* < 0.001, η^2^ = 0.09), ERD_ɑ_ (*F*_(2,173)_ = 20.817, *p* < 0.001, η^2^ = 0.19) and PD (*F*_(2,128)_ = 3.600, *p* = 0.03, η^2^ = 0.05), not for ISCR. Bonferroni corrected post hoc comparisons evidenced stronger ERS_θ_ in runs with low performance compared to those with middle (*t*_(173)_ = 2.627, *p* = 0.028) and high performance (*t*_(173)_ = 3.963, *p* < 0.001), and smaller PD compared to high performance level (*t*_(128)_ = −2.666, *p* = 0.026). ERD_ɑ_ was significantly lower in runs with low performance compared to those with both middle (*t*_(173)_ = −4.238, *p* < 0.001) and high (*t*_(173)_ = −6.155, *p* < 0.001) performance.

### 3.4 Predicting Performance With Physiological Features

Finally, the logistic regression including the individual performance level as a categorical variable (i.e., only low vs. high levels), and all the psychophysiological variables as predictors (i.e., ISCR, PD, ERS_θ_, ERD_ɑ_), was significant (*X* ^2^ = 27.434, *p* < 0.001). Interestingly, the significant variables predicting the difference between low vs. high performance were pupil diameter (*z* = −2.690, *p* = 0.007) and ERD_ɑ_ (*z* = −2.944, *p* = 0.003).

Finally, the linear regression confirmed this relationship also when including NF performance as a continuum (*F*_(4,116)_ = 7.057, *p* < 0.001, *R* ^2^ = 0.20). In agreement with the previous analysis, here again the significant variables predicting NF scores were pupil diameter (*t* = 2.026, *p* = 0.045) and ERD_ɑ_ (*t* = 3.629, *p* < 0.001).

## 4 Discussion

This study investigated the relationship between neurofeedback (NF) performance and a set of psychophysiological features—specifically, frontal theta event‐related synchronization (ERS), frontal alpha event‐related desynchronization (ERD), pupil diameter (PD), and phasic skin conductance response (ISCR)—during an EEG‐based motor imagery NF task in healthy adult participants. Although no significant group‐level improvement in NF scores was observed within a single session, our findings indicate that fluctuations in performance are systematically associated with distinct psychophysiological changes at both the group and individual levels.

As expected, significant differences for alpha‐beta band power were observed in motor (i.e., C3, Cz, C4) and sensorimotor (i.e., CP1, CP2) channels between rest and task blocks, consistent with prior literature on task‐related sensorimotor desynchronization (Lioi, Cury, et al. 2020; Neuper et al. 2006; Pfurtscheller et al. 2006). Frontal theta synchronization was also reliably observed during task blocks relative to rest, in line with research linking theta power to cognitive control, executive effort, and sustained attention (Clayton et al. 2015; Puma et al. 2018; Magosso et al. 2021). Importantly, frontal theta synchronization, alpha desynchronization, and pupil diameter were related to NF performance both within individuals and globally across all participants, suggesting their potential as dynamic markers of cognitive engagement during NF.

Frontal midline theta band power was elevated during task periods, but more interestingly, higher theta synchronization was associated with lower NF performance. This may suggest that beyond a certain threshold, elevated theta may reflect compensatory effort or cognitive overload rather than optimal engagement. This pattern mirrors findings by Puma et al. (2018), who reported that lower‐performing participants exhibited higher theta spectral power during a working memory task. Consistent with earlier studies (Klimesch 1999; Kramer 1991), theta power in this context may index cognitive effort or strain rather than efficient task execution.

In contrast, greater frontal alpha desynchronization was positively associated with NF performance. It is important to distinguish this finding from the sensorimotor alpha‐beta desynchronization used to generate the neurofeedback signal. During motor imagery, ERD of sensorimotor rhythms over central electrodes (i.e., Cz, C3, C4, CP1, CP2) reflects activation of the motor network and constitutes the neural feature typically used to control EEG‐based motor imagery neurofeedback (Cheyne 2013; Pfurtscheller and Neuper 2001). This pattern arises because imagined and executed movements recruit substantially overlapping neural substrates, particularly during kinesthetic motor imagery (Solodkin et al. 2004). In contrast, the frontal alpha desynchronization was measured independently of the feedback signal and is thought to reflect attentional allocation and workload rather than motor imagery itself (Clayton et al. 2015; Magosso et al. 2021; Puma et al. 2018; Vidulich and Tsang 2012). Therefore, our findings suggest that frontal alpha activity provides complementary information about the cognitive processes supporting successful neurofeedback performance, beyond the sensorimotor activity used to control the visual metaphor.

In line with our results, increased workload has been proposed to lead to frontal and parietal theta synchronization coupled with alpha desynchronization (Gevins 2000; Gevins and Smith 2003; Smith et al. 1999), indicating that both oscillatory patterns are sensitive to attentional demands. Puma et al. (2018) similarly reported that higher performers exhibited lower alpha spectral power, supporting a negative correlation between alpha power and task performance. Frontal alpha desynchronization thus appears to signal successful cortical engagement, whereas higher alpha power has been linked to disengagement and mind‐wandering (Compton et al. 2019), reinforcing the idea that alpha desynchronization supports task focus, while elevated alpha may signal attentional lapses. In our study, reduced frontal alpha (i.e., greater desynchronization) during high‐performance NF runs may suggest that participants were more focused and less prone to internal distractions (for example, mind‐wandering).

Taken together, these results reinforce the notion that theta and alpha oscillations reflect attentional load and engagement in opposite directions with respect to optimal NF performance: elevated theta may index compensatory or overloaded processing, whereas alpha desynchronization signals successful engagement and focus on task. In line with our results, Puma et al. (2018) had emphasized that efficient task execution is associated with moderated oscillatory responses rather than maximal activation, supporting our observation that spectral power in both bands is lower in higher performers.

Notably, frontal alpha desynchronization was not only associated with performance but, together with pupil diameter, emerged as significant predictors of the performance level (intended as a category as well as a continuum). Pupil diameter was significantly larger during task than rest conditions and it positively correlated with NF scores. This is consistent with Saeedpour‐Parizi et al. (2020), who identified pupil diameter as the most sensitive eye‐based biomarker of cognitive effort during goal‐directed tasks. Larger pupil diameter may reflect increased phasic activation of the locus coeruleus–noradrenergic system, facilitating optimal attentional states (Aston‐Jones and Cohen 2005). These findings extend prior evidence linking pupil dilation to cognitive load (Piquado et al. 2010), task engagement and performance (van den Brink et al. 2016), highlighting pupil diameter as a reliable, time‐sensitive marker of attentional allocation during the execution of cognitive tasks.

Phasic skin conductance responses exhibited a declining trend across runs—likely reflecting habituation to task demands—but did not relate to NF performance. This suggests that general arousal indexed by skin conductance responses fluctuates over time but is less directly linked to task‐specific engagement than markers of attentional control such as pupil diameter and EEG oscillations.

Importantly, analyses at the individual level revealed that theta synchronization, alpha desynchronization, and pupil diameter varied systematically in relation to individual changes in performance. Tracking performance at the single‐subject level is particularly critical given the large variability in NF responsiveness. Supporting this view, Bodala et al. (2016) showed that vigilance and engagement, as indexed by EEG and eye‐tracking parameters, can be enhanced by introducing appropriate task challenges. Real‐time monitoring of frontal theta/alpha oscillations and pupil metrics could thus enable adaptive NF protocols—for example, adjusting feedback difficulty or prompting short breaks when indicators of disengagement emerge. Interestingly, a recent study has proposed a model for monitoring cognitive engagement based on the previous 10 s of eye‐tracking and skin conductance signals while executing different cognitive tasks (Fragueiro et al. 2026). Our current results provide new input for the development of a model to monitor task engagement during NF training.

As a limitation in our study, we notice the lack of observable learning in brain activity regulation along the session. However, this is expected after a single session of NF, as NF improvements typically require multiple sessions. Nevertheless, we observed a trend toward performance improvement within the session, which is associated with concurrent changes in psychophysiological features.

In summary, this study highlights the relevance of multimodal physiological monitoring during neurofeedback training. Optimal NF performance appears to depend on a finely tuned interplay between neural oscillations indexing cognitive control and load (alpha and theta bands) and an autonomic marker of engagement (pupil diameter). Theta synchronization and alpha desynchronization are both sensitive to task demands but play functionally distinct roles: theta reflects compensatory cognitive effort or strain, while alpha reflects attentional allocation, potentially protecting against disengagement. These results support the development of personalized NF approaches that adapt to individual engagement profiles, which could improve training efficacy and reduce the proportion of non‐responders. Future NF systems may integrate these biomarkers to tailor training dynamically, maintaining participants in optimal engagement states.

## 5 Conclusion

In the present study we extracted EEG (alpha desynchronization and theta synchronization), pupil diameter and skin conductance responses while the participant was executing a motor imagery neurofeedback (NF) session, and we investigated the relationship between these physiological markers and changes in NF performance.

Our findings demonstrate that specific physiological indicators (i.e., alpha desynchronization, theta synchronization, and pupil diameter) track fluctuations in NF performance within individuals but also in general across the whole group. Thus, these physiological markers may be used to monitor engagement and predict performance during task execution. These results highlight the potential and encourage further research on combining EEG and peripheral measures to assess and dynamically adapt NF sessions to individual engagement profiles, optimizing attentional engagement during neurofeedback training.

## Author Contributions

**Agustina Fragueiro:** conceptualization, investigation, funding acquisition, writing – original draft, methodology, validation, visualization, writing – review and editing, formal analysis, data curation. **Claire Cury:** conceptualization, funding acquisition, writing – review and editing, project administration, resources, supervision, methodology, investigation. **René‐Paul Debroize:** investigation, software, methodology.

## Funding

This study was funded by INRIA Action exploratoire 2021 (EyeSkin‐NF) to C.C. and by the European Union—Next Generation EU, Mission 4, Component 2 (CUP: D73C24001280006) to A.F.

## Conflicts of Interest

The authors declare no conflicts of interest.

## Data Availability

Data is available under request to the authors. The complete dataset will be openly published by the end of the project.

## Endnotes

^1^

https://eeglab.org/tutorials/05_Preprocess/rereferencing.html.
